# Coping with the Forced Swim Stressor: Towards Understanding an Adaptive Mechanism

**DOI:** 10.1155/2016/6503162

**Published:** 2016-01-06

**Authors:** E. R. de Kloet, M. L. Molendijk

**Affiliations:** ^1^Division of Medical Pharmacology and Leiden Academic Center for Drug Research, Leiden University, Einsteinweg 55, 2333 CC Leiden, Netherlands; ^2^Division of Endocrinology, Leiden University Medical Center, Albinusdreef 2, 2333 ZA Leiden, Netherlands; ^3^Institute of Psychology, Leiden University, Wassenaarseweg 52, 2333 AK Leiden, Netherlands; ^4^Leiden Institute for Brain and Cognition, Leiden University Medical Center, Albinusdreef 2, 2333 ZA Leiden, Netherlands

## Abstract

In the forced swim test (FST) rodents progressively show increased episodes of immobility if immersed in a beaker with water from where escape is not possible. In this test, a compound qualifies as a potential antidepressant if it prevents or delays the transition to this passive (energy conserving) behavioural style. In the past decade however the switch from active to passive “coping” was used increasingly to describe the phenotype of an animal that has been exposed to a stressful history and/or genetic modification. A PubMed analysis revealed that in a rapidly increasing number of papers (currently more than 2,000) stress-related immobility in the FST is labeled as a depression-like phenotype. In this contribution we will examine the different phases of information processing during coping with the forced swim stressor. For this purpose we focus on the action of corticosterone that is mediated by the closely related mineralocorticoid receptors (MR) and glucocorticoid receptors (GR) in the limbic brain. The evidence available suggests a model in which we propose that the limbic MR-mediated response selection operates in complementary fashion with dopaminergic accumbens/prefrontal executive functions to regulate the transition between active and passive coping styles. Upon rescue from the beaker the preferred, mostly passive, coping style is stored in the memory via a GR-dependent action in the hippocampal dentate gyrus. It is concluded that the rodent's behavioural response to a forced swim stressor does not reflect depression. Rather the forced swim experience provides a unique paradigm to investigate the mechanistic underpinning of stress coping and adaptation.

## 1. Introduction

Validated animal models and tests are crucial for understanding the pathogenesis and treatment of mood and anxiety disorders [1]. This contribution is about the exposure of rodents to a forced swim stressor, which was originally designed by Porsolt et al. [2–4] to assess the antidepressant potential of drugs. The so-called forced swim test (FST, see Box 1) is based on the observation that when rats or mice are immersed in a beaker of water from where escape is not possible, they display a progressive increase in the frequency and duration of episodes of immobile floating after initial attempts to escape by swimming, struggling, climbing, or diving. In a retest the animals show the acquired immobility response almost immediately; the total time spent immobile and/or the duration of time until the transition from active to passive behaviour are the read-out parameters of this test. In mice a single session is often applied, which obviously excludes the retention of acquired immobility used in the test-retest design as criterion.

**Figure 1:**
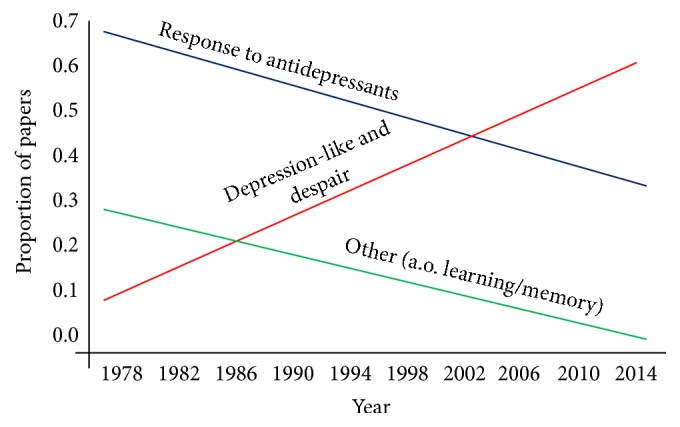
The figure shows the interpretation of the outcomes of the FST as a function of year of publication. The slopes that are plotted indicate that over the years more studies choose to label the outcomes from the FST as “depression-like behaviour” (the red line) and fewer as “antidepressant” properties of drugs (the blue line) or behavior such as “learned immobility” (the green line). For more information on these data we refer to Molendijk and de Kloet [8].

Porsolt's design of the FST was extremely productive for drug screening. The test appeared highly reproducible among different labs, lasted only 2 days, and was applicable for high-throughput [5–7]. However, an unfortunate aspect is the anthropomorphic interpretation of the rodent's progressive immobility during the FST as “lowered mood” or “despair” … and “giving up hope to escape,” which is highlighted as a depression-like phenotype [2–4, 6]. With the advent of mouse mutants carrying genetic modifications the FST was adopted as a rapid “animal depression” test. Hence, a dramatic increase occurred in the number of papers reporting in rodents the depressogenic effect of genes (see Figure 1), often in a context of early life adversity as well as later life acute or chronic exposure to stressors of all kind. In 1985 one paper per month was published that reported the results from the FST, today this number amounts to one per day [8]. For discussions of the rodent's forced swim performance as a measure for depression we refer to a series of excellent articles elsewhere [5–7]. For a critical evaluation of animal models for depression, see Nestler and Hyman [1].

In a recent commentary in* Psychoneuroendocrinology*, we presented an analysis of current interpretations of FST behavior [8]. The data for this analysis consisted of random samples of the 4,300 PUBMED listed papers in which the use of the FST was described. We found that the papers in which the FST was used to identify a depression-like phenotype amounted to around 2,020. Rapidly declining over the years (now in total 1,980 papers) was Porsolt's original FST application of the test for identification of a compound's antidepressant potential. In about 820 papers the “depressogenic” effect of stress was studied. We further estimated that in 320 studies the FST was used for phenotyping genetic mouse mutants. Finally, in about 300 papers, and rapidly declining over the years (see Figure 1), the progressive immobility was interpreted as a learning process. These 300 studies demonstrated that the outcome of the FST in the test-retest paradigm could be altered by interfering with acquisition, consolidation, and retention of the immobility response [9, 10].

In this contribution to the* Many Faces of Stress *issue, the progressive immobility that is acquired during the FST is presented as a passive behavioural style of the rodent to cope with the situation that escape from the beaker is not possible.

In the first section of this paper we will sketch the transition of active (swimming) to passive (immobility) behaviour as an adaptive learning process that contributes to survival by conserving energy, likely evolving from millions of years of evolution. We will highlight recent reports on the transition from active to passive behaviour and how this adaptive response is stored in memory. It appears that glucocorticoids as well as antidepressants are capable to affect the ability of rodents to consolidate the learned immobility response [9, 11, 12].

In the second section we summarize the corticosteroid receptor balance concept of health and disease [13] and discuss its implications for immobility learning and memory storage. This includes the selection of the appropriate coping style involving cognitive flexibility and executive dopaminergic functions [14–16], pharmacological experiments to identify the brain sites of mnemonic action of the corticosteroids [17, 18], and a possible epigenetic mechanism as discovered by Reul [19]. We also will discuss the effect of a chronic stress history on the rodent's performance in the FST. We conclude, in the third section, with the notion that synthesis is possible according to the knowledge gained on corticosteroid action in limbic brain and dopaminergic executive functions. Hence, the rodent's response to an acute forced swim stressor provides an excellent opportunity to investigate the mechanism underlying stress coping and adaptation that contributes to survival.

## 2. Forced Swim

### 2.1. The Forced Swim Stressor

It is of interest to read the original articles of Porsolt et al. [2] and of Hawkins et al. [4], and the subsequent discussion between the authors of these papers. Hawkins et al. [4] agreed with using the FST as innovative antidepressant screening tool but dismissed Porsolt's notion that immobility in the FST represents despair. Hawkins et al. [4] noted by carefully monitoring the switches between swimming, headshaking, struggling, diving, climbing, and floating that the progressive immobility at the end of the 15 min initial test is an adaptive response* “without the energy expenditure required in swimming.*” At retest, 24 hours later, a similar level of immobility is immediately resumed. Moreover, the rats appeared at retest less emotional, observed as a lower amount of emotional defecation, which according to Hawkins supports the idea that* “… having been rescued on day 1, the rats were less fearful on day 2.*” In reply Porsolt maintains the position that the immobility response in the FST measures lowered mood and despair and acknowledges that the procedure is “*not a model for depression in the rat*” but reflects “*… some aspects of depressed mood.*” In addition, Porsolt mentioned that also electroshocks, the treatment of choice when more conventional methods fail in alleviating the symptoms of depression, readily led to decreased immobility scores in the FST mimicking the effects that are observed after administration of pharmacological antidepressants.

In 2009, Castagné and colleagues stated that “…* the FST is not a model for depression because the dependent variable is the response to the acute forced swim stressor rather than the phenotype of the animal*” [20]. Since there is no sign or symptom of depression modeled in the FST it lacks* face validity*. Also construct* validity* is absent since the pathogenesis of depression is a slow process that is often, but not always, precipitated by the inability to cope with the stress of life [21]. Indeed in many experiments animals are subjected to a chronic stress paradigm with or without a genetic mutation and then the FST is used incorrectly to model depression. The FST shows* predictive validity* where it concerns the testing of antidepressant potential of compounds. This validity criterion is without evidence of the mechanism* how* antidepressants affect the switch from active to passive behaviour. Rather, the antidepressants that disrupt immobility in the FST acutely take several weeks before they are clinically effective in a depressed patient [22] suggesting that also this predictive validity of antidepressant action in the FST gives little insight into any pathogenic mechanism. Besides, antidepressants affect multiple functional domains beyond mood, including memory and appetite [22] that potentially could have an effect on FST performance.

There are many* false positives*. For instance drugs like amphetamine, which is not an antidepressant, enhance locomotor activity and prevent the switch to immobility [3, 4]. Also the GABA-A agonist muscimol, barbiturates, benzodiazepines, and anticholinergic agents have been coined as false positives (see De Pablo et al. [23] for an overview). Rats engaged in physical exercise, in humans regarded as being “antidepressant” [24], show increased immobility and secrete increased amounts of corticosterone but also are more resilient [19]. Physical exercise thus could be regarded as a* false negative* in this context. Widely described antidepressant agents of the SSRI type [22] likewise could be regarded as a false negative [25]. Furthermore, animals that are familiar to the test are more immobile [26] just as animals that are exposed to water of 19°C in the initial test which became more immobile if the water at retest was 25°C rather than the original 19°C [19]. Finally, in a brief report on 2 experiments O'Neill and Valentino [27] ruled out that the extent of escapability from the beaker of water reflected a measure of despair: the immobility response during retest was identical irrespective of the presence of an escape option. They also demonstrated that the FST is not a learned helplessness model.

### 2.2. Active and Passive Coping

As students of the FST, more than 20 years ago, we have performed experiments to examine the role of stress hormones in the acquisition and retention of the rodent's response to the acute swim stressor. We observed the switches between the different behaviours towards longer periods of immobility and that acquired immobility was retained at the 24 hr retest. The retention of acquired immobility may last as long as 4 weeks [28]. In our line of reasoning the switch to immobility behaviour “…* is a successful passive behavioural strategy.*” [29], which appeared affected by antisense manipulation of the glucocorticoid receptor (GR) in the hippocampus if performed at least six hours before the initial test.

Immobility in the FST was also interpreted as passive coping [30]. Since coping has a positive connotation “* … dealing effectively with something difficult*” (Oxford Dictionary; http://www.oxforddictionaries.com, accessed September 5th, 2015) this qualification is somewhat at variance with labeling the passive coping style as a symptom of depression [30, 31]. Cabib and colleagues [10, 18] formulated after a series of elegant experiments using stress-susceptible DBA mice in a test-retest design the hypothesis that “*immobility is the result of extinction-like inhibitory learning involving all available escape responses due to the inescapable/unavoidable nature of the FST experience.*” Other qualifications are “*… that immobility is beneficial in preventing the rats from sinking*”:* rodents that float longer probably live longer* [32]. Although some of these explanations suffer from anthropomorphism (e.g., despair, depression-like), the switch between the different behavioural (coping) responses towards increased immobility shows what actually is observed when an animal deals with the forced swim stressor.

Fascinating novel technology currently allows real-time measurement of the transition between active and passive behavioural states with simultaneous* in vivo* electrophysiological recordings. Using these techniques, striking correlates were found between the activity of specific medio prefrontocortical (mPFC) and mesolimbic dopaminergic circuits and the transition between active and passive behavioural states at the 24 h FST retest [15, 30, 31]. These transitions were interpreted as representing elements of neuronal encoding and a subsequent decision-making process that is reflected in the behaviours observed in the FST. Moreover, using optogenetic activation of specific mPFC and midbrain dopaminergic subcircuits (the former projecting to the dorsal raphe nuclei) the behavioural transitions towards immobility were induced suggesting a causal relationship [30].

Tye et al. [31] tested selective ventral tegmental area (VTA) A9 dopaminergic neurons after viral transfection with an enhanced halorhodopsin that shows upon stimulation hyperpolarization and thus dopaminergic inactivation. They found upon AVT inhibition increased immobility in the FST, while locomotor responses were not affected. Moreover, causal relationships of specific circuit activations during the 24 hr retest occurred in parallel with other putative “depression” tests such as the FST, tail suspension, and sucrose preference test [31]. Interestingly, the same authors demonstrated that acquired immobility, enhanced by a history of chronic stress exposure, could be reversed within seconds by light stimulation of the same dopaminergic neurons transduced with channelrhodopsin-2 [31] to achieve the desired neuronal activation. However, opposite results were reported by Chaudhury et al. [33]; see for discussion of these studies Lammel et al. [15]. Noteworthy is that some of the brain circuits linked to passive-active transitions were also identified (i.e., the nucleus accumbens and medial frontal cortex) as targets for the immediate antidepressant effects of deep brain stimulation [34].

Accordingly, these data obtained by optogenetic manipulation of the VTA dopaminergic neurons provide strong evidence for a causal relationship with forced swim performance. The VTA dopaminergic circuit and its mesocortical and mesolimbic branches have however complex afferent and efferent pathways that operate in multiple feedback loops [35]. Cabib and Puglisi-Allegra [16] have built a compelling case that enhanced tonic mesoaccumbens dopamine activity supports the expression of active stress-induced coping styles, while inhibition of dopamine release is required for passive coping (immobility) in the FST. The latter passive behaviour occurs when a stressful condition is appraised as inescapable and/or uncontrollable.

The pioneering research by the Grace group (see for an overview [14]) focused in particular on the balance in afferent pathways from the ventral hippocampus and basolateral amygdala which was found to regulate a spontaneous single-spike firing pattern of the VTA dopamine neurons. This “tonic” pacemaker is driven by the excitatory outflow of the ventral subiculum hippocampus via the nucleus accumbens, ventral pallidum pathway (see [14]). In agreement with Cabib and Puglisi-Allegra [16] and Tye et al., [31] also Grace [36] noted that uncontrollability of the stressor suppressed the mesoaccumbens pathway, while promoting the expression of a passive coping response.

### 2.3. Consolidation of Acquired Immobility

**Figure 2:**
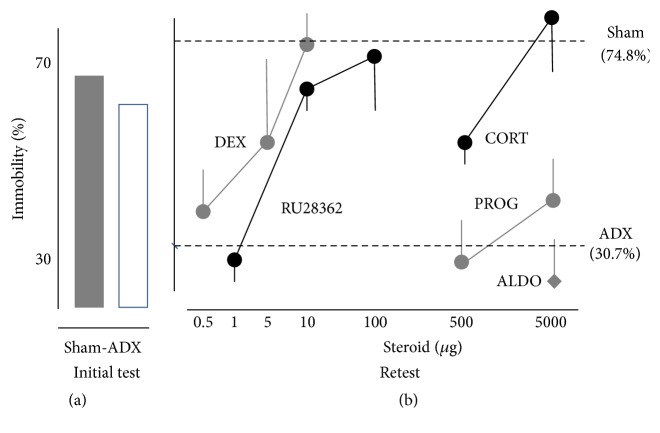
(a) Left panel shows % immobility during the last 5 min of the 15 min initial test of SHAM and adrenalectomised (ADX) animals. (b) Right panel shows the effects of various steroids, given 15 min after the initial swimming exposure, on retention of acquired immobility of ADX rats during the 5 minutes retest period. Data are expressed as mean ± the standard error of the mean as % immobility time. Dashed lines represent the % immobility of SHAM (74,8%) and ADX (30,7%) rats. Fifty ADX and 33 sham rats were used in these experiments. Six animals were used per different dosages of dexamethasone, cortisol, RU38362, and progesterone. Post hoc comparisons following a significant ANOVA revealed that the groups treated with DEX (5 and 10 *μ*g), RU28362 (10 and 100 *μ*g), and cortisol (500 and 5000 *μ*g) differed significantly from ADX but not from sham rats. For more information on these data we refer to [11].

Jefferys et al. [12] and Veldhuis et al. [11] reported that rats, adrenalectomised 1 week before the initial test, showed levels of immobility that were similar to controls. However, at retest the immobility response was not retained. The naturally occurring glucocorticoid corticosterone and the synthetic glucocorticoids dexamethasone and RU2362 given subcutaneously 15–60 min after the initial test reinstated retention, while mineralocorticoids and progesterone had no effect (see Figure 2). As expected the antiglucocorticoid RU486 given prior to the initial test interfered with the glucocorticoid-induced retention of acquired immobility. Interestingly, removal of the adrenal medulla, secreting adrenaline and opioids, only transiently interfered with retention, which could be restored by administering synthetic enkephalin analogs [37]. Subsequent experiments showed that also thyroid hormone and glucose [38, 39] are effective, suggesting interplay between endocrine and metabolic factors during retention of immobility. It is likely that the action of these factors in promoting immobility also would promote conservation of the energy needed to prolong survival, which is actually one of the lessons for sailors in the* essentials of sea survival* [40].

De Pablo et al. [9] reported a number of well-controlled experiments clearly demonstrating that antidepressants interfere with the consolidation process in the FST (see Table 1). Using an automatic recording procedure to assess mobility, they demonstrated that during the forced swim experience the amount of immobility increased with repeated experience. Exposing the rats to a cylinder with increased water depth led to decreased immobility. This is counterintuitive because more “despair” would have been expected. Finally, antidepressants given after the initial test interfered with consolidation of the acquired immobility response as measured from the rat's performance at retest 24 hr later. Since the protein synthesis inhibitor anisomycin had similar effects as the antidepressants it is evident that effects measured in the FST retest monitor memory storage of the behavioural response acquired at the initial test. The paper by De Pablo et al. [9] is a “must read” for everyone who uses the test-retest design of the FST.

### 2.4. Conclusion

Exposure to the forced swim stressor induces a profound response of the sympathetic nervous system, the HPA axis, and also of a variety of neurotransmitter circuits (e.g., dopamine, serotonin, GABA) in the brain [31, 41–43]. In particular the VTA-A9 dopaminergic circuitry has received much attention because of its role in mediating the stress response and in the pathogenesis of stress-related depression and psychosis. Indeed, the mesoaccumbens dopaminergic circuit is important for coping with the forced swim stressor. In the forced swim test transitions proceed progressively from active to passive coping styles culminating in prolonged periods of immobility. These transitions serve as measure in the mouse single trial FST as well as in the retest. They can be evoked by optogenetic stimulation of the VTA dopaminergic neurons. Dopaminergic activity is under control of afferent inputs from limbic areas, that is, amygdala and hippocampus. All these limbic-forebrain areas are targets for corticosteroid hormones released under stress.

## 3. Corticosteroid Action and Stress

### 3.1. Corticosteroid Receptors and Action

The naturally occurring glucocorticoids, cortisol in man and corticosterone in man and rodent, are collectively abbreviated as CORT here. CORT regulates energy metabolism and controls the stress response. The hormones, secreted by the adrenals as end product of the HPA axis, coordinate in rhythmic fashion the needs in circadian regulations from food intake to allocation of energy resources. CORT also mediates coping with stress in a manner that the hormones prevent the initial reactions to a stressor from overshooting [44, 45]. These actions exerted by CORT are mediated by mineralocorticoid receptors (NR3C2, MR) and glucocorticoid receptors (NR3C1, GR) [46–49]. MR and GR regulate gene transcription as nuclear receptors and occur also as membrane variants that are engaged in rapid nongenomic membrane actions [50–52].

The MR and GR have different characteristics [48, 53–56]. First, MR expression is abundant in limbic structures, notably the hippocampus, amygdala, lateral septum, and regions of the prefrontal cortex where it is colocalized with the ubiquitously expressed GR. Second, nuclear MR has a tenfold higher affinity for CORT than GR and is therefore always substantially occupied, while GR only becomes occupied after stress and at the ultradian or circadian peaks in circulating CORT. Third, MR in most brain regions is nonselective: CORT and aldosterone have high affinity and also deoxycorticosterone and progesterone bind, the latter as a competitive antagonist [57].

The actions mediated by MR and GR are complementary: in some cells and circuits opposing and elsewhere synergizing [59–61]. On the cellular level MR maintains and enhances the excitatory tone [62, 63]. Activation of the MR membrane variant (which has a lower affinity to CORT than the nuclear MR) by stress stimulates the release of glutamate, which subsequently downregulates the presynaptic Glu2/3 receptors [50, 60, 64, 65]. With rising steroid concentrations CORT suppresses, via the GR, the excitability which is transiently raised by excitatory stimuli [66]. In nongenomic fashion GR promotes the postsynaptic release of endocannabinoids, which inhibit transmitter release presynaptically [67].

**Figure 3:**
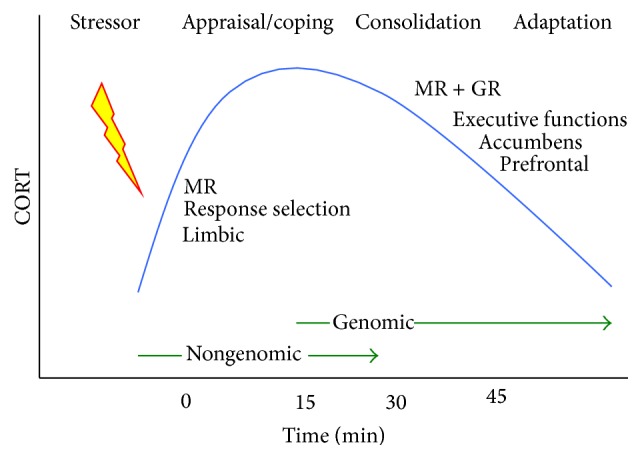
Corticosterone action during processing of stressful information. Increasing corticosterone concentration induced by a stressor initially activates MR modulating appraisal processes and immediate coping and then progressively activates also nuclear GR to reallocate energy to circuits underlying consolidation and retention of the experience in the memory [58]. For this purpose MR and GR mediate in complementary fashion the action of corticosterone in hippocampus and amygdala from decision-making and cognitive flexibility to executive functions in prefrontal brain regions, as is mediated by the mesolimbic dopaminergic system innervating the nucleus accumbens. Adapted from de Kloet et al. [13]. MR is mineralocorticoid receptors; GR is glucocorticoid receptors.

On the behavioural level MR and GR mediate distinct functions in the processing of stressful information (see Figure 3). The MR mediates a tonic action on the activity of the HPA axis [68] and is important during the onset of the stress reaction because it regulates anticipation, appraisal, response selection, and thus decision-making processes in coping with novel stressful situations. These are all functions linked to the limbic network [13, 69]. When the stress response develops and CORT concentrations rise, the GR becomes progressively occupied which allocates additional energy resources towards the more executive frontocortical functions [58]. Primarily the action of CORT, mediated by the GR, is aimed to promote behavioural adaptation which terminates the stress reaction. At the same time the outcome of the coping process is stored in memory for future use [70, 71]. When the stress reaction subsides the ultradian rhythm resumes allowing to maintain a state of stress responsiveness.

The GR and MR thus have complementary functions in the processing of stressful information. This has led to the formulation of the Corticosteroid Receptor (CoRe) Balance hypothesis which states that “*upon imbalance of MR:GR-regulated limbic-cortical signaling pathways, the initiation and/or management of the neuroendocrine stress response becomes compromised. At a certain threshold this may lead to a condition of HPA-axis dysregulation and impaired behavioural adaptation, which can enhance susceptibility to stress-related neurodegeneration and mental disorders*.” [13, 54, 55, 68, 69].

### 3.2. CORT Receptors and FST

Three types of experiments that link CORT receptors to the typical FST behavior will be discussed here. First, it appears that the forced swim stressor itself affects the expression of the CORT receptors differentially. It was shown that the acute stressor induced the expression of MR in the hippocampus as early as 8 hr postinjection and the effect appeared maximal at 24 hr after exposure to the forced swim stressor, at the immunoreactive protein level as well as with radioligand binding [72, 73]. This MR induction depended on CRF, since exogenous CRF induced and CRF antagonist blocked the stress-induced increase in MR. Finally, the CRF-induced MR synthesis appeared functional since in prior forced swim exposed rats antimineralocorticoids were much more effective in disinhibiting the stress-induced HPA-axis activity [72]. Over a period of several weeks the hippocampal MR is profoundly downregulated after exposure to chronic stress, however [74, 75]. This downregulation of MR was prominent in socially defeated mice that showed increased passive coping [76].

Second, GR activation by dexamethasone administration in the low *μ*g range to adrenalectomised animals immediately after the initial 15 min forced swim exposure reinstated dose-dependently the deficit in retention of acquired immobility during the 5 min retest 24 hr later. This effect of dexamethasone in the ADX rats can be prevented by prior subcutaneous administration of the RU486, or other GR antagonist(s) in doses of 1 and 10 mg/kg [17, 77]. Intracerebroventricular adminstration of the GR antagonist to intact rats immediate before the initial test attenuated at retest the retention of acquired immobility in a 100 000 lower dose than needed after systemic administration. In a separate experiment one week later the same low ng dose of RU486 icv increased secretion of CORT [17]. Thus, functional GR is neccesary for retention of acquired immobility.

**Figure 4:**
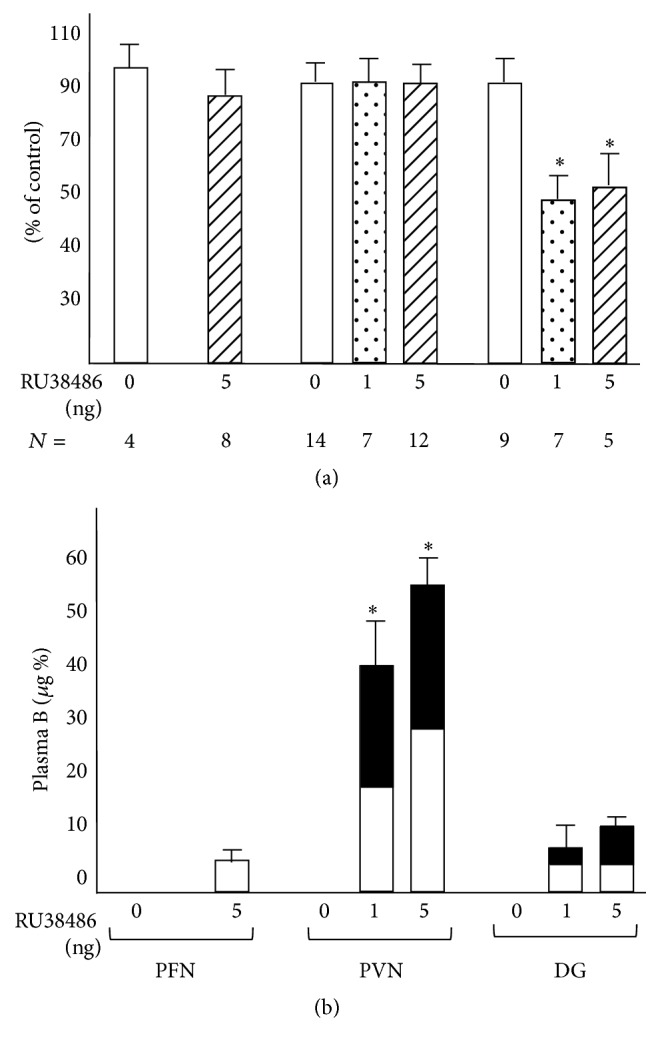
(a) Effect of local intracerebral injection of RU38486 at 5 min prior to initial test on retention of acquired immobility (top) and (b) plasma CORT level (bottom). Behavioural data are expressed as percentage (mean ± the standard error of the mean) of the value observed in rats injected with vehicle. Endocrine data are expressed as micrograms CORT per 100 mL plasma. Open areas represent control injections and closed areas the CORT levels after administration of RU38486. The number of animals is indicated in the figure. The figure comes from de Kloet et al. [17]. ^*∗*^ *P* < .01 as compared to controls. PFN is thalamic parafascular nucleus; PVN is hypothalamic paraventricular nucleus; DG is dentate gyrus. Adapted and reprinted with permission from Karger.

Figure 4 (adapted from [17]) shows that only 1 ng of RU486 administered in the dentate gyrus is sufficient to impair consolidation of the immobility response. Similar injections in the nucleus parafascicularis and paraventricular nucleus were ineffective, but the GR blockade in the paravenricular nucleus triggered a profound CORT response. Promegestone did not interfere with the RU486 action ruling out a role for the antiprogestin properties of the antagonist. The selective mineralocorticoid antagonist RU28318 was not active excluding, as expected, a role of MR in retention of the passive coping style. The exclusion of MR in memory consolidation is further reinforced by the observation that replacement of the ADX rats with a high dose of CORT occupying both receptor types reinstated the memory deficit of the ADX rats, while a lower dose, mainly occupying MR, did not.

If GR was blocked with RU486 given systemically at 6 hr (but not at 1 hr) prior to the initial test the percentage of immobility was decreased during the initial 15 min test. This decrease was already present in the first 5 min episode and persisted in the retest 24 hr later [29]. A similar result was obtained if the synthesis of GR in the dentate gyrus was inhibited by bilateral infusion of 18-mer antisense phosphorothioate oligodeoxynucleotide targeted to GRmRNA 6 hours prior to testing [29]. Likewise daily treatment* plus* a 1 hr pretreatment with GR antagonists also suppressed immobility at pretest, but this design did not include a retest [78, 79]. Reduced immobility was also observed at the initial and retest after metapyrone, which blocks the synthesis of adrenal CORT [80]. Interestingly, these experiments involving blockade of GR or reduction of adrenal output leave MR available for CORT action, supporting indirectly a role of MR in coping with the forced swim stressor.

### 3.3. MR and GR Function in Acquisition, Consolidation and Retention of Immobility

Colelli et al. [18] demonstrated different levels of immobility learning in DBA/2J and C57Bl/6J mice. In the first experiment both strains showed that the immobility scores in the 10 min initial test were retained in the 5 min retest 24 hr later. Immobility scores in the C57 mice were much higher than in the DBA. In the second experiment it was shown that the immobility performance of the DBA mice correlated, 50 min after initial test, with enhanced expression of cFos in the dorsal striatum, while in the dorsal hippocampus the immediate early gene altered in parallel with C57 immobility. This enhanced activity in the hippocampus aligns with the greater context and spatial memory performance of the C57 that coincides with more CORT output than observed in the DBA strain [81].

Hippocampal MR has a crucial role in the switch from spatial declarative learning towards caudate stimulus response (habit) learning [82]. In the circular hole board test, naive male mice locate with a hippocampal-associated spatial strategy an exit hole at a fixed location flagged by a proximal stimulus. However, if exposed to a stressful context, close to 50% of the mice switched to habit learning associated with hypertrophy of the caudate and atrophy of the hippocampus under chronic stress conditions [83]. Pretreatment with an MR antagonist did prevent the switch towards the stimulus-response strategy [84]. These findings are consistent with evidence that during stress a MR-dependent increase in amygdala connectivity underlies the shift from hippocampal spatial learning to striatal stimulus response or habit learning [82]. With regard to coping with the forced swim stressor, MR antagonists administered prior to the initial test are predicted, therefore, to affect immobility learning in the FST. Indeed, two studies showed that administration of the MR antagonist spironolactone in rats and mice ([85, 86], resp.) reduced the amount of immobility of the animals.

In a series of studies Reul and his colleagues developed the concept that CORT secreted during the initial acute swim stress experience triggers in the dentate gyrus a signaling pathway that activates an epigenetic process underlying increased consolidation and retention of newly acquired stressful information [28]. This mechanism concerned convergence of stress-induced NMDA and GR signaling pathways causing in a distinct and sparse neuroanatomical pattern of dentate gyrus neurons histone modifications, chromatine remodelling, and immediate early gene activation [87–89]. Genetic deletion of specific components (i.e., MSK1/2) in this pathway appeared to prevent the retention of acquired immobility. The significance of this newly identified pathway has been expanded to the role of epigenetics in Morris maze learning, while revealing new interesting twists in their significance for memory consolidation [90].

### 3.4. The Effects of Chronic Stress

Processing of the forced swim stressor has both physical and psychological components [41]. Physical stressors such as pain, cold, heat, and water immersion each have their inputs to directly stimulate the common final pathway to activation of the sympathetic nervous system and the HPA axis. Psychological or psychogenic stressors are processed in higher brain regions, potentially using multiple circuits [91, 92] illustrating* the Many Faces of Stress*. However, severe acute stressors can have long-term consequences as well and are of obvious significance as triggers to precipitate an altered phenotype. The acute forced swim stressor has been used for this purpose alone or in combination with another acute single restraint stress exposure [74, 93]. Such animal models for chronic stress exposure also are based on various protocols, for instance exposure of the animals repeatedly during several days to unpredictable stressors, repeated exposure to the same stressor, or daily social defeat, sometimes with a history of early life adversity [94, 95].

The A9 mesolimbic-cortical dopaminergic circuitry is highly responsive to acute and chronic stressors. The responsiveness of this circuitry depends on reciprocal hippocampal ventral subiculum excitatory and amygdala inhibitory inputs [14] including a feedback loop to the A9, but also to the habenular nucleus, dorsal raphe nucleus, basal amygdala, and ventral hippocampus [15]. The circuit has an important function in social and goal-directed behaviour, motivation, pleasure, and reward and is richly endowed with GR. Rodents exposed to repeated social defeat by aggression of a dominant animal develop enduring social aversion and increased anxiety as most prominent behavioural adaptations caused by a CORT-enhanced positive dopaminergic feedback loop. Antiglucocorticoid or GR deletion selectively from the dopaminoceptive neurons reinstated social behaviour linking stress resiliency with dopaminergic tone [96, 97]. As mentioned above, in this circuit correlations were found between circuit activity and the passive-active behavioural transitions during forced swim exposure, which could also be induced optogenetically [15, 30, 31].

Chronic restraint stress induces rapid changes in histone regulation in the hippocampus [98]. Chronically stressed animals likewise show profound changes in neuroendocrine regulations due to an altered phenotype of the CRH neurons expressing much more vasopressin as cosecretagogue [99]. Such chronically stressed animals also display dramatic chromatin reorganizations in CORT brain targets. This altered reorganization becomes apparent only after challenging the stressed individual with an additional acute forced swim stressor. Mice with a stress history exposed to forced swim for 15 min showed much more responsive genes 1 hr later in the hippocampus, and these are particularly genes involved in chromatin modification, epigenetics, and the cytokine/NF*κ*B pathway. The change in some of these genes (e.g., BDNF and GR) persisted for several weeks [94]. Besides, these genes are related to cognitive processes [100] presumably underlying immobility learning in the FST [9, 10].

Interestingly, similar cytokine/NF*κ*B genomic changes were observed after repeated social defeat [101]. The network also showed overlap with the genomic response to CORT applied to rats with a restraint stress history, in this case restricted to the dentate gyrus only [102]. Moreover, in the controls 26 different CORT responsive gene ontology (GO) terms were enriched, whereas this number was only 6 in the stressed group. One highly responsive gene network revealed by this procedure is the mammalian target of rapamycin (mTOR) signalling pathway, which is critical for different forms of synaptic plasticity [103] that may underlie the processes of learning and memory [104].

### 3.5. Conclusion

GR is expressed abundantly in the ascending A9 mesolimbic and cortical dopaminergic projection innervating frontocortical and nucleus accumbens target regions, while the limbic structures, notably the hippocampal CA1 and CA2 neurons as well as the dentate gyrus neurons, are richly endowed with both MR and GR. The receptors seem to be involved in acquisition and retention of the immobility response. For* acquisition*, pharmacological blockade of GR by systemic RU486 administration and locally by GR knockdown in the dentate gyrus 6 hr* prio*r to the initial test* decreased* the amount of passive behaviour, which was retained in the retest 24 hr later. This effect apparently overrides the small increase in immobility noted after local knockdown of GR in the infralimbic frontocortical dopaminergic target [105]. The* consolidation *and* retention *of the passive behavioural response are promoted after GR activation in the dentate gyrus by an epigenetic mechanism that involves a GR-glutamatergic pathway. However, any blockade of the GR in the limbic regions will result in more dominance of CORT actions via the MR, which could participate in the appraisal, response selection, and immediate coping ability [18]. Such a role of MR has been firmly established in other behavioural paradigms [84, 106].

## 4. Perspectives

An inevitable consequence of* the Many Faces of Stress* is the discussion centered around the seminal question: “*What is stress?*” For this reason one of the stress pioneers, Levine [107–109], turned to use an operational definition: “*Stress is defined as a composite multidimensional construct in which three components interact: (i) the input, when a stimulus, the stressor, is perceived and appraised, (ii) the processing of stressful information, and (iii) the output, or stress response. The three components interact via complex self-regulating feedback loops with the goal to restore homeostasis through behavioral and physiological adaptations.*”

What happens during processing of the forced swim stressor in the black box of the rodent's brain? The studies by Cabib and coworkers, that are summarized in Cabib and Puglisi-Allegra [16] and Campus et al. [10], point to a genetically determined switch between hippocampus and striatal circuits as a determinant in the choice of behavioural style to cope with the forced swim. Such an MR-induced switch previously was observed in other behavioural paradigms as well [82, 110, 115]. This finding calls for a role of CORT acting via MR during stress, which has been shown crucial for appraisal, immediate coping, response selection, and behavioural flexibility. MR was discovered in electrophysiological studies to mediate fast and rapidly reversible membrane actions of CORT in hippocampus and amygdala [50, 51]. Stress levels of the hormone enhance the frequency of miniature excitatory postsynaptic potentials (mEPSP) in hippocampal CA1 pyramidal neurons, indicating enhancement of glutamate excitatory outflow from the hippocampus [60, 64, 65, 112].

**Figure 5:**
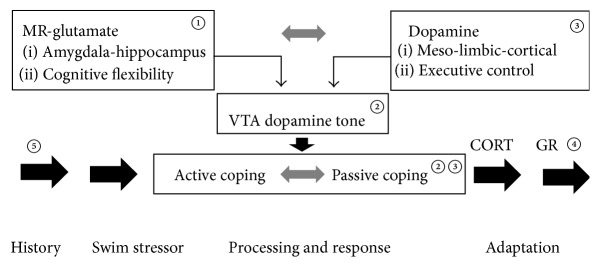
Hypothesis: processing of the forced swim stressor. ① The stressor is perceived and appraised and the appropriate coping style is selected depending on flexibility of amygdala-hippocampus-striatal connectivity, which is controlled by CORT via limbic MR. ② This action of CORT mediated by MR modulates the hippocampal excitatory outflow through enhanced glutamate transmission driving the spontaneous activity of the VTA-A9 neurons and active coping. ③ When time elapses energy is allocated to more executive functions governed by mesocortical and prefrontal circuitry attenuating mesoaccumbens dopamine activity causing a switch from active to passive coping with the inescapable forced swim stressor. ④ The coping response is stored in memory for future use by a mechanism activated by stress-induced levels of CORT acting through the GR in the hippocampal dentate gyrus. ⑤ The information processing during the forced swim is affected by stress history as can be deducted from altered genomic expression in the hippocampus. This hypothesis is based on the following references: [14–19, 28, 30, 31, 36, 50, 51, 58, 64, 82, 84, 94, 102, 110–114]. For more information we refer to the main text of this paper. CORT is corticosterone; MR is mineralocorticoid receptors; GR is glucocorticoid receptors; VTA is ventral tegmental area, A9 dopaminergic neurons.

Grace [36] highlights an important role of the afferent circuits in processing of contextual and emotion-loaded information that operate reciprocally from the hippocampal ventral subiculum and the basolateral amygdala in regulating A9 dopaminergic activity [14]. This afferent excitatory control would be driving the behavioural expression of accumbens dopamine release as can be demonstrated by coping with the forced swim stressor. The optogenetic experiments by Tye et al. [31] indeed show an executive role for the mesolimbic dopaminergic system in the transitions between active and passive coping, while Warden et al. [30] and Cabib and Puglisi-Allegra [16] showed evidence for implication of the mPFC circuitry. Then, after the initial test the preferred passive coping style is stored in memory for future use by a GR dependent process in the hippocampal dentate gyrus [17, 19]. We refer to Figure 5 for some of the elements that may be involved in processing the forced swim stressor in the brain.

How hippocampal MR participates through enhanced excitatory transmission in the transition between active and passive behaviour needs to be investigated. That the subsequent rise in CORT after forced swim activates GR to promote consolidation and retention of the coping style in the memory is firmly established. For this purpose an epigenetic mechanism underlying consolidation of the acquired immobility has been identified in the hippocampal dentate gyrus [19]. Furthermore, a history of chronic stress downregulates in particular hippocampal MR [76, 93], introducing a bias, which is reflected by altering the genomic response in hippocampus and dentate gyrus to forced swim [94]. Part of this response is mimicked by CORT action with consequences in the dentate gyrus neurogenic niche [102, 113].

Floating has been a criterion in the past to judge the witchcraft outcome of forced swim [114], but today it is in use to label a rodent as being depressed. In fact, the number of research papers that intentionally used the FST to assess a depression-like phenotype has shown a dramatic increase in recent years, now amounting to almost one paper per day [8]. Hence, this anthropomorphic interpretation of coping with the forced swim stressor is remarkable, since alternatively the forced swim experience provides a unique challenge to investigate how information processing occurs to achieve stress adaptation.

The use of acquired immobility to diagnose depression in a rat should not be encouraged. What we do encourage is to use the forced swim stressor in research on the mechanism of coping and adaptation that counts to understand an evolutionary-conserved energy-sparing survival mechanism of passive coping with an apparent inescapable/uncontrollable situation.

## Figures and Tables

**Box 1:**
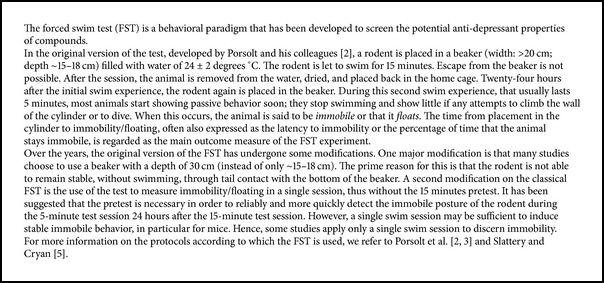
**Box 1: **The forced swim test.

**Table 1:** The effect of a single dose of 25 mg/kg of imipramine administered one hr before or 15 min after training on day 1 on rat mobility in the forced swim test.

Injection time	*N*	No. of impulses, day 1	% no. of impulses, day 2
Saline	7	409.85 ± 48.79	42.63 ± 11.50
15 min after	7	420.14 ± 45.93	94.39 ± 8.38^*∗*^
1 hour before	6	421.66 ± 39.75	93.06 ± 16.44^*∗*^

^*∗*^Statistical significance (at *P* < .01) with respect to the saline group.

Adapted and reprinted from De Pablo et al. [9] with permission from Elsevier B.V.
